# The Inescapable Effects of Ribosomes on In-Cell NMR Spectroscopy and the Implications for Regulation of Biological Activity

**DOI:** 10.3390/ijms20061297

**Published:** 2019-03-14

**Authors:** David S. Burz, Leonard Breindel, Alexander Shekhtman

**Affiliations:** Department of Chemistry, University at Albany, State University of New York, 1400 Washington Ave., Albany, NY 12222, USA; dsburz@albany.edu (D.S.B.); lbreindel@albany.edu (L.B.)

**Keywords:** Ribosome, mRNA, rRNA, Thioredoxin, Adenylate kinase, Thymidylate synthase, Dihydrofolate reductase, cross-correlated relaxation, protein interactions, protein structure-function, enzyme activity, enzyme kinetics, NMR spectroscopy

## Abstract

The effects of RNA on in-cell NMR spectroscopy and ribosomes on the kinetic activity of several metabolic enzymes are reviewed. Quinary interactions between labelled target proteins and RNA broaden in-cell NMR spectra yielding apparent megadalton molecular weights in-cell. The in-cell spectra can be resolved by using cross relaxation-induced polarization transfer (CRINEPT), heteronuclear multiple quantum coherence (HMQC), transverse relaxation-optimized, NMR spectroscopy (TROSY). The effect is reproduced in vitro by using reconstituted total cellular RNA and purified ribosome preparations. Furthermore, ribosomal binding antibiotics alter protein quinary structure through protein-ribosome and protein-mRNA-ribosome interactions. The quinary interactions of Adenylate kinase, Thymidylate synthase and Dihydrofolate reductase alter kinetic properties of the enzymes. The results demonstrate that ribosomes may specifically contribute to the regulation of biological activity.

## 1. Introduction

For the past two decades, in-cell NMR spectroscopy has been used to investigate the structure, dynamics and interaction surfaces of proteins inside living cells [1,2,3,4,5,6,7]. In recent years a few intrinsically disordered proteins, IDPs, such as alpha-synuclein [5], Pup [8], and FG repeats [9,10] and folded proteins, such as GB1 [11] and SOD1 [12], have provided in-cell NMR spectra of satisfactory quality for quantitative analysis. However, the in-cell NMR spectra of most folded proteins are poorly resolved when employing the pulse sequences typically used to study proteins in vitro [13]. Binding interactions between the target protein and intracellular constituents result in macromolecular complexes with apparent molecular weights on the order of 1 MDa [14,15] that scale linearly with intracellular viscosity and are consistent with in vitro apparent molecular weights of 300–400 kDa [16,17]. As larger species tumble more slowly the result is a widespread broadening of in-cell NMR spectral peaks [14,16]. These specific low-affinity interactions, dubbed quinary interactions, are omnipresent due to the high concentration of interacting species, which provide the chemical energy for binding interactions [18,19,20].

To be detectable by in-cell NMR, target proteins have to be present in-cell at concentrations ≥10 µM [13,21,22,23]. What intracellular species exist at sufficiently high concentrations to give rise to protein quinary structures? Genomic DNA is too large (>10 MDa), has too low an abundance and is largely inaccessible in eukaryotic cells [24]. Proteins, with an average molecular mass of ~50 kDa [25], and tRNAs, ~20 kDa [24], will not form complexes of the size observed. That leaves mRNA, 100–500 kDa, and rRNA, up to 5 MDa, as the most likely candidates for the interacting complement to protein quinary structural complexes.

The intracellular concentrations of mRNA have been estimated to range from 2–20 µM in prokaryotes and 50–500 nM in eukaryotes [24]. Ribosome concentrations in prokaryotes and eukaryotes can exceed 10 µM and 1 µM, respectively [25]. These concentrations are high enough to ensure a wide range of binding interactions with target proteins that are introduced into or over-expressed in cells. The ubiquity of these interactions forms the bedrock for quinary structural states that represent the primary conformation adopted by most proteins in cells [26].

Over the past few years, work in our laboratory has suggested that RNA, in particular ribosomes, plays a major role in establishing protein quinary structures [14,26]. This conclusion is in general agreement with mass spectroscopic studies of mRNA- and ribo-interactomes [27,28,29,30] in which hundreds of eukaryotic proteins bound to either mRNA or ribosomes were identified and did not possess obvious RNA binding motifs. Such observations have provided a glimpse of insight into the physical complexity of quinary interactions [31,32,33,34]. Additional evidence suggests that the RNA-bound quinary state may have a different activity than the unbound state of the protein studied in vitro [26,35]. In this article, we will review the evidence for implicating RNA as an integral component that interacts with folded proteins to establish quinary structure (Table 1) and show that the biological activity of a protein is altered when bound to ribosomes.

## 2. Protein-RNA Interactions Broaden Target Protein NMR Spectra

**Figure 1:**
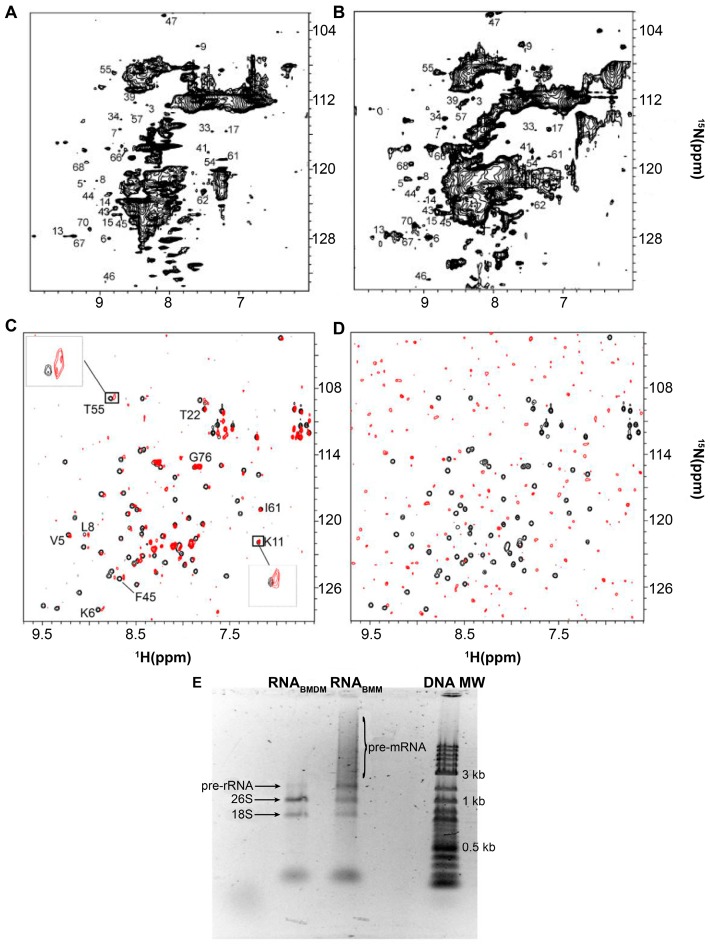
Total cellular RNA alters in vitro spectra of Ubiquitin, Ubq. (**A**) In-cell ^1^H-^15^N heteronuclear single quantum coherence, HSQC, NMR spectra of [*U*- ^15^N] Ubq in *P. pastoris* after 24 h of methanol induction and (**B**) 48 h of methanol induction. (**C**) Overlay of the in vitro ^1^H-^15^N HSQC spectra of 10 μM [*U*- ^15^N] Ubq in the absence (black) and presence (red) of 30 mg/mL of RNA_BMM_ and (**D**) 30 mg/mL of RNA_BMDM_. Insets in panel C show a broadening of selected residues of free Ubq (black) due to the interaction with RNA_BMM_ (red). (**E**) RNA from yeast cells grown with methanol, RNA_BMM_, contains an amount of pre-mRNA and pre-rRNA larger than that of RNA from cells grown with a methanol/dextrose carbon source, RNA_BMDM_. DNA MW indicates molecular weight markers. The numbers in panels A, B and C indicate some of the peak assignments. Panels A and B are adapted from Bertrand et al. (2012) [40]. Panels **C**, **D** and **E** are adapted from Majumder et al. (2016) [35].

Bertrand et al. [40] noted that changing the carbon source during growth of the yeast *Pichia pastoris*, *P. pastoris*, altered the intracellular distribution of the uniformly labeled overexpressed target protein Ubiquitin, [*U*- ^15^N] Ubq, from *Saccharomyces cerevisiae, S. cerevisiae*. The in-cell ^1^H-^15^N heteronuclear single quantum coherence, HSQC, the spectrum of Ubq acquired from cells grown in methanol displays many broadened and missing peaks suggesting that Ubiquitin interacts with large intracellular complexes (Figure 1A). The dispersion of the detectable peaks indicates that Ubiquitin is well folded, but background signals from small ^15^N labeled metabolites, which dominate the central region of the spectra, impede high-resolution analysis. For cells grown on methanol and dextrose, the spectrum is undetectable. The in-cell NMR spectrum of Ubq collected 48 h post-induction (Figure 1B) contains stronger signals suggesting that a larger fraction of the population is free to tumble inside the cells. By overexpressing Ubq for a very long period most of the binding sites become saturated allowing free Ubiquitin to be observed.

To determine if these results are due to Ubq–RNA quinary interactions, in vitro ^1^H-^15^N HSQC NMR spectra were collected on [*U*- ^15^N] Ubq in the absence and presence of total RNA prepared from yeast cells grown in buffered methanol medium, RNA_BMM_, and in buffered methanol/dextrose medium, RNA_BMDM_ [35]. The effect of RNA on the HSQC NMR spectra was dramatic. Consistent with in-cell observations [40], in the presence of 30 mg/mL of RNA_BMM_ a subset of Ubq crosspeaks were broadened (Figure 1C) suggesting a specific interaction between the labeled target and RNA [35]. In the presence of 30 mg/mL of RNA_BMDM_ all of the spectral peaks disappeared (Figure 1D).

There were conspicuous differences between the two RNA preparations: RNA_BMM_ contained preprocessed large ribosomal and mRNA that was absent from RNA_BMDM_ (Figure 1E). Control HSQC spectra collected in the presence of up to 50 mg/mL of chondroitin sulfate, a glycosylate linear polyanion, did not affect the basis spectrum suggesting that the Ubq-RNA interaction is specific [35]. The conclusion was that Ubq quinary interactions were regulated by the total cellular RNA content, which was, in turn, regulated by the growth conditions, and that the affinity of the interaction increased in the presence of fully processed RNA. The use of total RNA preparations successfully recapitulated in-cell observations and provided an in vitro platform for further investigating the role of RNA in promoting and maintaining quinary structural states.

## 3. Resolving Target Protein Bound to RNA

The problem of widespread target protein in-cell HSQC NMR signal broadening is not limited to yeast. Indeed, virtually all proteins display these spectral characteristics in both mammalian and bacterial cells [14,41,42]. The absence of widespread line broadening in early experiments performed in *E. coli* was due to the fact that the overexpressed proteins leaked out of the cells during in-cell NMR experiments [43] or overexpression of labeled target exceeded 100 µM [13], which is ≥10 times greater than the estimated dissociation constant of 1–10 µM for target protein quinary interactions. At this concentration, in-cell NMR signal intensity is enhanced by a population of the unbound protein resulting in a greater number of sharper spectral peaks. At lower intracellular concentrations binding of the labeled target is stoichiometric. Due to the high concentration of RNA present in cells, line broadening is inevitable for proteins expressed at physiological levels. To ascribe biological relevance to the structural interactions revealed by in-cell NMR spectra it was necessary to adopt methods for detecting large labeled targets at or near physiological concentrations.

Peak broadening is due to the formation of massive quinary interaction complexes. The large MW species tumble more slowly and exhibit a reduced transverse relaxation time for the NMR signal, T2 [44,45]. T2 depends on the rotational diffusion of a molecule in solution and is inversely related to the rotational correlation time, τ_c_ [45]. Shorter T2 values cause the NMR signal from larger molecules to decay more rapidly and lead to extensive line broadening [44]. This effect is pronounced in the case of folded proteins where all nuclei experience global rotation. Notable exceptions include intrinsically disordered proteins, IDPs, and protein with intrinsically disordered regions, IDRs [46]. These proteins lack persistent secondary or higher structure, possess fast local dynamics, and fail to interact with intracellular constituents resulting in in-cell spectra that are much sharper than those typically observed for folded proteins [47].

HSQC and heteronuclear multiple quantum coherence, HMQC, pulse sequences [45], originally used for in-cell NMR spectroscopy [48], use insensitive nuclei enhanced by polarization transfer, INEPT, pulse sequences to transfer magnetization from protons to heteronuclei, but the efficiency of INEPT deteriorates with decreasing T2 [49]. Transverse relaxation-optimized spectroscopy, TROSY, which suppresses transverse nuclear spin relaxation in heteronuclear NMR experiments during evolution and acquisition cycles [50] in combination with ^15^N-edited cross relaxation-induced polarization transfer, CRINEPT, NMR spectroscopy [51,52], which increases the efficiency of magnetization transfers between heteronuclei, can be used to improve the resolution and sensitivity of in-cell NMR experiments for large complexes. Further improvement in sensitivity can be achieved by optimizing the CRINEPT-like magnetization transfer delay time in the ^1^H-^15^N CRINEPT-HMQC-TROSY pulse sequence, and by employing REDuced PROton density (REDPRO) labeling [53], which exchanges alpha and beta protons of amino acids for deuterons to minimize proton relaxation. The resulting in-cell ^1^H-^15^N CRINEPT-HMQC-TROSY pulse sequence when applied to [*U*- ^2^H, ^15^N] labeled target protein yields a spectrum in which most of the target protein crosspeaks are resolved.

**Figure 2:**
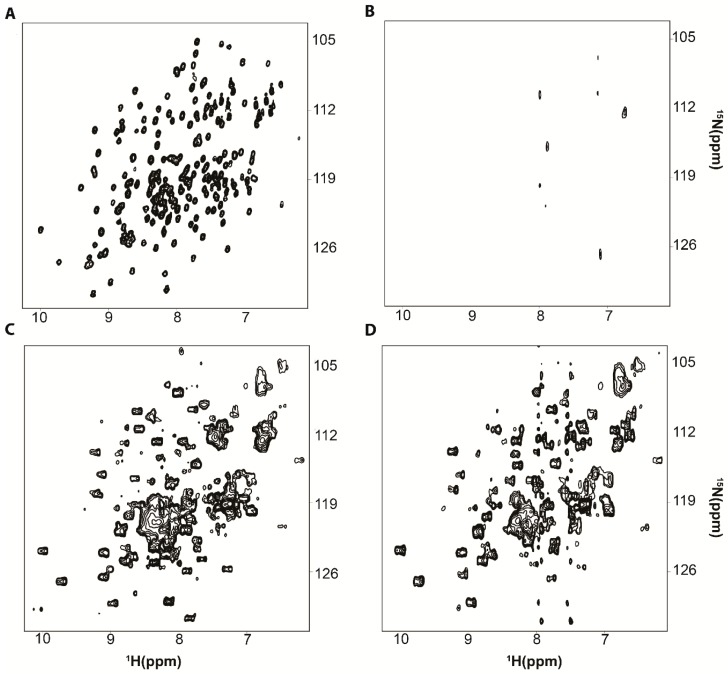
^1^H-^15^N CRINEPT-HMQC-TROSY improves in-cell NMR spectral resolution. (**A**) Lysate HSQC spectrum of [*U*- ^15^N] Adenylate kinase, ADK. (**B**) In-cell ^1^H-^15^N HSQC spectrum of [*U*- ^15^N] ADK overexpressed for 16–18 h. (**C**) In-cell ^1^H-^15^N CRINEPT-HMQC-TROSY spectrum of [*U*- ^2^H, ^15^N] ADK overexpressed for 16–18 h. (**D**) In vitro ^1^H-^15^N CRINEPT-HMQC-TROSY spectrum of 10 µM purified [*U*- ^2^H, ^15^N] ADK in the presence of 2.5 µM ribosomes. The peak shapes in **C** and **D** arise from a population of free and bound species due to the high concentration of target protein (>100 µM).

The improvement in spectral resolution is shown in Figure 2 for *Escherichia coli*, *E. coli*, Adenylate kinase, ADK. Using the ^1^H-^15^N HSQC pulse sequence the in vitro ^1^H-^15^N correlation spectrum is well-resolved (Figure 2A) but cannot be observed in *E. coli* cells (Figure 2B). Majumder et al. 2015 [14] utilized ^1^H-^15^N CRINEPT–HMQC–TROSY NMR to investigate uniformly ^2^H and ^15^N labeled, [*U*- ^2^H, ^15^N], ADK in *E. coli* and was able to resolve many of the target protein peaks (Figure 2C). Similar results were obtained by using ^1^H-^15^N CRINEPT-HMQC-TROSY NMR to examine bacterial Thioredoxin, Trx and FK506 binding protein, FKBP, in *E. coli*, and human Ubq in HeLa cells [14]. Most importantly, the ^1^H-^15^N CRINEPT–HMQC–TROSY NMR spectrum of 10 µM [*U*- ^2^H, ^15^N] ADK collected in vitro in the presence of 2.5 µM ribosomes exhibited broadened peaks that largely coincide with the in-cell spectrum (Figure 2D). This observation supports the idea that RNA, specifically ribosomes in the case of ADK, are the binding complement that gives rise to quinary interactions.

## 4. Target Protein-RNA Complexes Exhibit Megadalton Apparent Molecular Masses

Optimizing the CRINEPT transfer delay time, *T_opt_*, can provide an estimate of the apparent molecular weight of the target protein. Theoretically [52,54] *T_opt_* is a solution of

(1) $$R_{c}\left[sinh\left(2R_{c}T_{opt}\right)\right]+\pi J_{NH}\left[sin\left(2\pi J_{NH}T_{opt}\right)\right]\;=2R_{H}\left[sinh^{2}\left(R_{c}T_{opt}\right)+sin^{2}\left(\pi J_{NH}T_{opt}\right)\right]$$

where *R_c_* is the relaxation rate resulting from the cross-correlation between ^15^N–^1^H dipole–dipole coupling and amide proton chemical shift anisotropy, *R_H_* is the transverse relaxation rate of the amide protons and *J_NH_* is a scalar ^15^N–^1^H coupling constant. *R_c_* and *R_H_* are related to the rotational correlation time, *τ_c_*, by *R_c_* = 1.7*τ_c_B*_o_ and *R_H_* = *τ_c_*(0.8*B*_o_^2^ + 1.7), where *τ_c_* is in nanoseconds, *B*_o_ is the strength of the magnetic field in gigahertz and *R_c_* and *R_H_* are in seconds. In combination with the Debye–Stokes–Einstein relation [55]

(2) $$\tau_{c}=(4\pi \eta R_{H}^{3})/3kT.$$

where *η* is the viscosity of the medium, *R_H_* is the hydrated radius of the protein, *k* is the Boltzman constant and *T* is absolute temperature, the apparent molecular weight, MW_app_, of protein inside cells, bound to RNA, or in viscous glycerol solutions can be estimated. Solving T_opt_ for a range of *τ_c_* values will yield Stokes radii that can be used to approximate MW_app_ by assuming a generic value for the partial specific volume of a protein equal to 0.73 cc/g [51].

**Figure 3:**
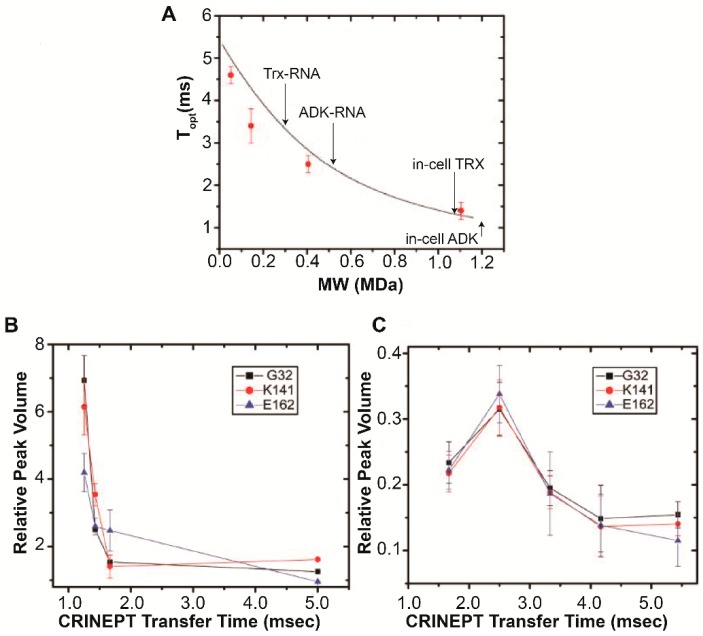
Optimizing the CRINEPT transfer delay time yields in-cell target protein apparent molecular weights. (**A**) The dependence of *T_opt_* on the apparent in-cell molecular weight, MW_app_ at 700 MHz. *T_opt_* was experimentally determined at 5 °C (red symbols) by using 100 μM [*U*- ^2^H, ^15^N] Trx dissolved in 10 mM potassium phosphate buffer, pH 6.5, containing 30, 65, 75, and 85% (*w*/*w*) d_5_-glycerol with corresponding viscosities of 4, 34, 92, and 343 cP, respectively [56]. The MW_app_ of *E. coli* ADK and Trx in-cell and in vitro in the presence of total *E. coli* RNA, uncorrected for intracellular viscosity, are indicated. (**B**,**C**) The relative volumes of the G32, K141 and E162 peaks in the ^1^H-^15^N CRINEPT-HMQC--TROSY spectra of [*U*- ^2^H, ^15^N] ADK collected in-cell (**B**) and in vitro at 20 µM in the presence of 50 µg of total RNA (**C**) are plotted against CRINEPT transfer delay times. In (**B**) an in-cell value of 1.2 ms was assigned because shorter transfer delay times interfere with CRINEPT pulses and limit the ability to acquire data. An endogenous tryptophan indole amide peak in the in-cell spectra was used as a reference. Panels (**A**–**C**) are adapted from Majumder et al. (2015) [14].

Data showing the dependence of *T_opt_* on the MW_app_ of *E. coli* Trx, measured in vitro with an increasing amount of glycerol, which restricts the rate of tumbling, is shown in Figure 3A [14]. The experimental data agree well with the theoretical curve generated using Equation (1). *T_opt_* was measured for *E. coli* ADK, MW 23.5 kDa, and found to be ≤1.2 ms (Figure 3B); the lack of a maximum in the in-cell *E. coli* buildup curve implies that the apparent molecular weight is ≥1.2 MDa. Transfer times shorter than 1.2 ms interfere with CRINEPT pulses and limit the ability to collect data. In vitro in the presence of total *E. coli* RNA *T_opt_* was 2.5 ms (Figure 3C), which corresponds to an apparent molecular weight, MW_app_, of ~0.4 MDa. Because ADK was present in molar excess over total RNA, the resolved MW_app_ reflects a population of free and RNA-bound ADK. Correcting for an intracellular viscosity of 3–4 cP, yields an in-cell MW_app_ of ~1.4 MDa. *E. coli* Trx, MW 11.8 kDa, exhibited a *T_opt_* of 1.3 ms in-cell, which corresponded to an MW_app_ of ~1.1 MDa, and in the presence of total *E. coli* RNA the uncorrected MW_app_ was ~0.3 kDa (Figure 3A), which translates to an in-cell MW_app_ of ~1.1 MDa. Given that the approximate MW of an *E. coli* ribosome is 1.3 MDa [25], MW_app_ for the target proteins observed in-cell and in vitro in the presence of total RNA are consistent with the formation of protein-ribosomal complexes.

## 5. RNA-Mediated Quinary Interaction Surfaces

### 5.1. Adenylate Kinase

The interacting surfaces of a target protein in-cell can be determined by using STructural INTeraction, STINT, NMR [4,57,58,59], which quantitates the changes in individual crosspeaks between the free and bound conformations. The signal from target protein surface residues is altered when engaged in binding interactions. For quinary interactions, the changes in chemical shift and/or intensity between in vitro or lysate target protein crosspeaks are compared to those observed in-cell to identify the quinary interaction surface. Further changes in those surfaces in response to stimuli can be analyzed by using singular value decomposition, SVD, which distinguishes concentration-dependent from concentration-independent changes in crosspeaks over time as the concentration of the stimulus increases [58,60]. STINT-NMR was used to investigate the quinary structure of ADK and Trx, and the changes in quinary structure in response to ribosomal-binding antibiotics [14,36].

**Figure 4:**
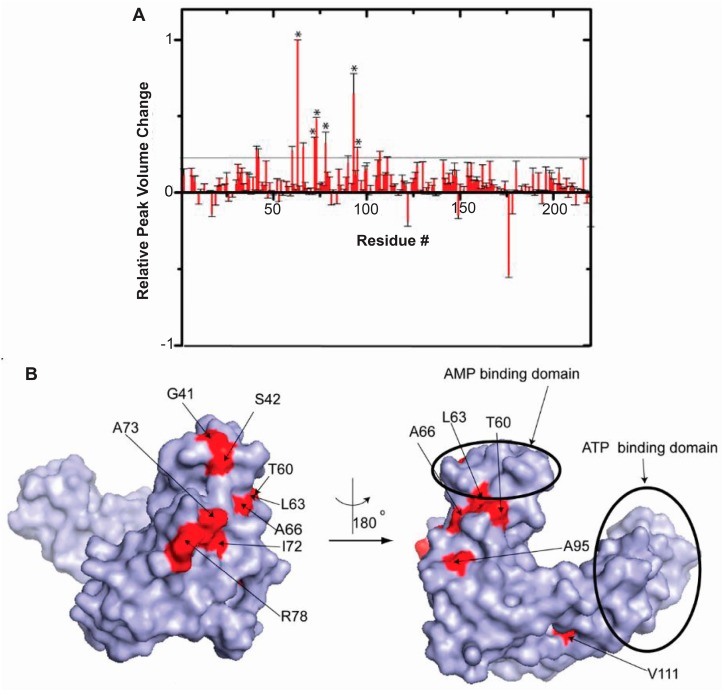
ADK quinary interaction surface does not block the active sites. (**A**) Relative changes in in-cell ^1^H-^15^N CRINEPT–HMQC–TROSY peak intensities of [*U*- ^2^H, ^15^N] ADK residues due to ribosome-mediated quinary interactions. The threshold line delineates residues whose NMR peaks undergo significant broadening. Residues that are affected by the interaction of ADK with total RNA are indicated with asterisks. (**B**) Residues involved in quinary interactions (red), mapped onto the molecular surface of ADK (Protein Data Bank, PDB, entry 4AKE), lie in the CORE domain of ADK. Panels (**A**,**B**) are adapted from Majumder et al. (2015) [14].

ADK catalyzes the transfer of a phosphate from ATP to AMP to create two ADP molecules [61]. In the absence of bound substrate, the enzyme exists in an open conformation in which the ATP and AMP binding domains are maximally separated; substrate binding reorients the domains closer together resulting in a closed conformation [62,63]. The ^1^H-^15^N CRINEPT-HMQC-TROSY spectrum of *E. coli* ADK collected in *E. coli* cells [14] indicates an open conformation in agreement with in vitro observations. The spectral broadening was characteristic of intermediate exchange, implying an interaction dissociation constant between 1–10 µM. The chemical shifts of residues involved in domain closure were unchanged showing that macromolecular crowding does not perturb the tertiary structure of the enzyme. A subset of peak intensities was broadened in the in-cell spectrum of ADK relative to what was observed in vitro or in lysates (Figure 4A). The residues that undergo the most dramatic changes in intensities in-cell define the quinary interaction surface (Figure 4B). This surface lies proximal to the AMP binding region in the CORE domain leaving the active sites of ADK unaffected and free to bind ATP and AMP.

**Figure 5:**
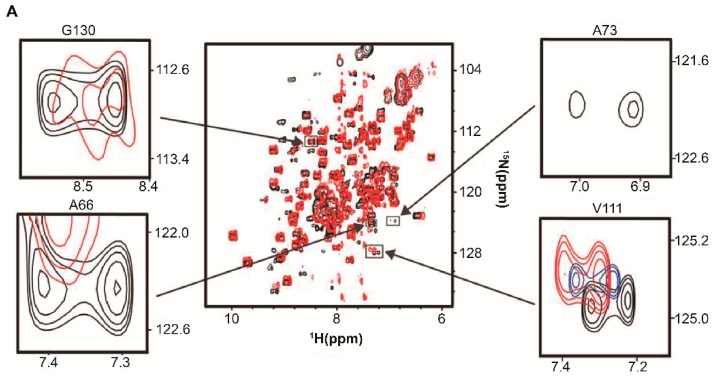

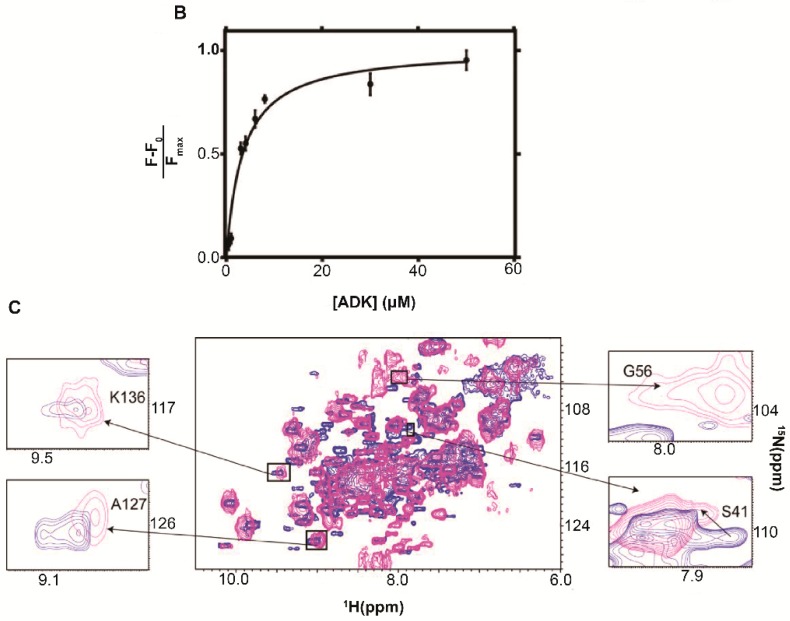
Quinary interactions of ADK in *E. coli*. (**A**) (Center) Overlay of in vitro ^1^H-^15^N CRINEPT-HMQC-TROSY spectra of 10 μM [*U*- ^2^H, ^15^N] ADK without (black) and with 2.5 μM ribosome (red). Surrounding panels show overlays of individual residues including in-cell NMR peaks (blue). (**B**) Fluorescence titration of 0.5 μM ribosome with ADK. Tryptophan fluorescence was measured at an emission wavelength of 350 nm by using an excitation wavelength of 280 nm. Curve fitting to a single site-binding isotherm yielded a K_d_ of 3.7 ± 0.4 μM. F_o_ is the fluorescence in the absence of ADK, and F_max_ is the maximum fluorescence of the ADK−ribosome complex. Fluorescence titration experiments were performed in triplicate. (**C**) Overlay of the in-cell ^1^H-^15^N CRINEPT-HMQC-TROSY spectra of [*U*- ^2^H, ^15^N] ADK in the absence (blue) and presence (magenta) of 100 μg/mL chloramphenicol. K136 and A127 (left insets) in chloramphenicol treated cells exhibit chemical shift changes consistent with ATP bound ADK; G56 and S41 peaks (right insets) exhibit chemical shift changes consistent with AMP bound ADK. Panels A and B are adapted from DeMott et al. (2017) [26]. Panel (**C**) is adapted from Majumder et al. (2015) [14].

The exact nature of the interaction was further clarified in vitro by using NMR (Figure 5A) and fluorescence titration (Figure 5B) to measure the binding of ADK to ribosomes. ADK was found to bind to ribosomes with a K_d_ of 3.7 ± 0.4 µM. The quinary contact surface identified by in-cell NMR therefore likely represents the ADK-ribosomal interface. To further interrogate the relationship between ADK and ribosomes, chloramphenicol, which binds to the large ribosomal subunit and increases the intracellular concentration of ATP [64], was introduced into *E. coli* and the resulting spectral changes in the [*U*- ^2^H, ^15^N] ADK spectrum were analyzed.

The addition of chloramphenicol perturbed the cellular equilibrium between ATP, ADP and AMP and dramatically altered the in-cell NMR spectrum of ADK. Changes in chemical shifts consistent with ATP- and AMP-bound ADK were observed (Figure 5C). The spectrum was similar to that observed in vitro with 3 mM ATP and 200 μM AMP, and consistent with a closed conformation of ADK. Collectively the results suggest that ribosomes may regulate the activity of ADK directly through quinary interactions, which may alter the affinity of the enzyme for ATP, or indirectly by altering the concentration of free ATP available for binding.

### 5.2. Thioredoxin

**Figure 6:**
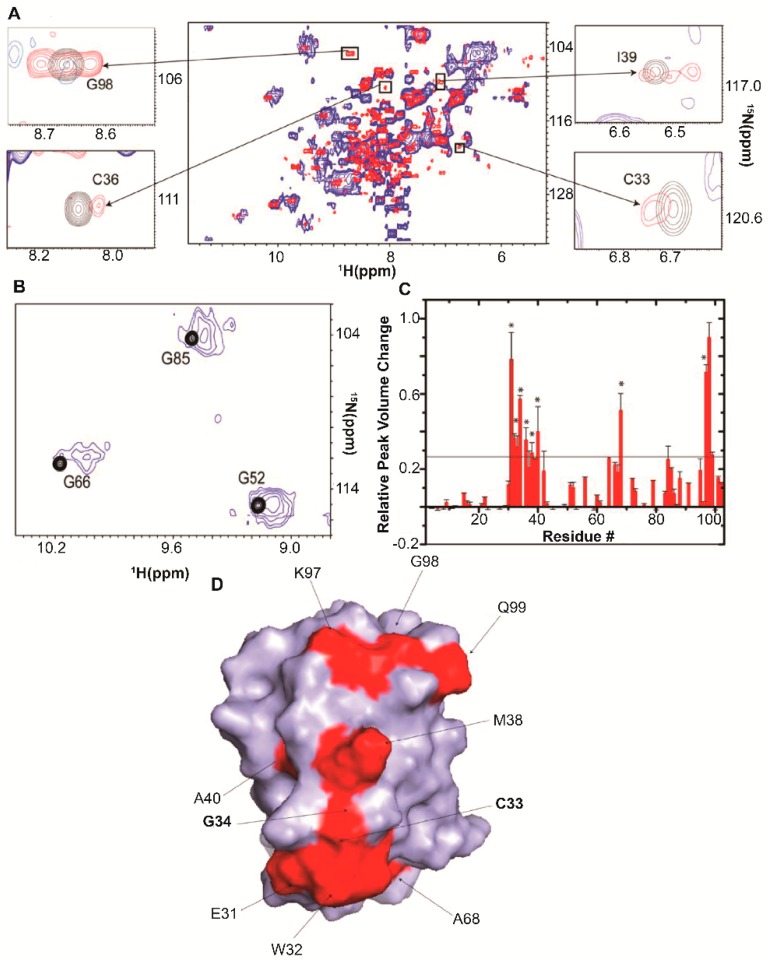
Quinary interactions of Trx in *E. coli*. (**A**) Overlay of the in-cell ^1^H-^15^N CRINEPT–HMQC–TROSY spectra of [*U*- ^2^H, ^15^N] Trx (blue) and that of the cellular lysate (red). The insets show overlays of the boxed regions of the in-cell spectrum (blue) and the corresponding regions of the ^1^H-^15^N CRINEPT–HMQC–TROSY spectrum of lysate (red) and the ^1^H-^15^N HSQC spectrum of purified Trx in 10 mM potassium phosphate buffer (pH 6.5) (black). The intensities of the C33, C36, I39, and G98 peaks, residues involved in quinary interactions, are broadened in-cell. (**B**) Overlay of the ^1^H-^15^N CRINEPT–HMQC–TROSY spectrum of [*U*- ^2^H, ^15^N] Trx in *E. coli* (blue) with crosspeaks from the ^1^H-^15^N HSQC spectrum of purified [*U*- ^2^H, ^15^N] Trx in 10 mM potassium phosphate buffer, pH 6.5 (black). G52, G66, and G85 exhibit broad in-cell peaks characteristic of multiple conformations of Trx in fast exchange on the NMR time scale, implying that the quinary interactions are inherently transient and dynamic. (**C**) Relative changes in in-cell ^1^H-^15^N CRINEPT–HMQC–TROSY crosspeak intensities of [*U*- ^2^H, ^15^N] Trx residues due to quinary interactions. The horizontal threshold differentiates residues whose NMR peaks undergo significant broadening. Residues annotated with asterisks are also affected in total RNA-bound Trx. (**D**) Residues involved in the quinary interactions (red) are mapped onto the molecular surface of Trx (PDB entry 1X0B); active site residues, C33 and G34, are in bold. The figure is adapted from Majumder et al. (2015) [14].

*E. coli* Trx, is a 12 kDa protein with redox activity that maintains a reducing environment inside the cell by means of active site cysteines. The ^1^H-^15^N CRINEPT-HMQC-TROSY spectrum of [*U*- ^2^H, ^15^N] *E. coli* Trx collected in *E. coli* cells [14] exhibited broad peaks at positions close to those observed in cell lysates (Figure 6A). The in-cell concentration of Trx was ~300 μM. Some of the in-cell NMR resonances exhibit two maxima, corresponding to fast and slow transverse relaxing components of crosspeaks (Figure 6B), suggesting that free cytosolic Trx is in exchange with a complex inside the cells. Despite this heterogeneity, only a subset of residues was broadened (Figure 6C). The results indicate that the quinary interaction surface of the molecule overlaps with the CGPC motif active site and adjacent regions (Figure 6D).

To determine if RNA is a component of Trx quinary interactions, ^1^H-^15^N CRINEPT-HMQC-TROSY spectra of 15 µM [*U*- ^2^H, ^15^N] Trx were collected in the presence of 30 mg/mL of both *E. coli* and *S. cerevisiae* total RNA [14]. The indole NH of W29 exhibited the same downfield shift in the in vitro RNA-bound and in-cell NMR spectra, while the indole NH of W32 and backbone amide peaks of E31, C33, C36, K37, I39, and A40 were broadened in a manner similar to the quinary interaction observed in-cell. Treating a mixture of purified Trx and total *E. coli* RNA with RNase A yielded a pool of small RNAs and nucleotides. If RNA oligonucleotides act as ligands, RNase treatment would increase the fraction of RNA-bound Trx due to the increase in the molar concentration of total RNA. Indeed, changes in the ^1^H-^15^N CRINEPT-HMQC-TROSY spectrum indicated an increase in the population of bound Trx and a reduced, ~20 kDa, MW_app_ both of which are expected to result from oligonucleotide binding. Total RNA and RNase-treated total RNA perturbed the same subset of peaks, indicating a specific quinary interaction surface for Trx.

To identify the RNA complement to Trx quinary interactions ^1^H-^15^N CRINEPT-HMQC-TROSY spectra of 150 µM [*U*- ^2^H, ^15^N] Trx were acquired in the absence and presence of 10 µM ribosomes [26]. No peak broadening was observed implying that there was no specific interaction between Trx and ribosomes. In studies of the mRNA interactome, the eukaryotic homologue of Trx was shown to bind to mRNA [27,65,66,67]. The conclusion was that the Trx-RNA interaction previously identified was likely mediated by mRNA.

The putative Trx-mRNA interactions provided an opportunity to test whether quinary structures can be indirectly affected through mRNA-ribosome interactions, specifically through the influence of ribosome binding antibiotics. Ribosome inhibition depends on how the antibiotic is bound: binding to the small, 30S, ribosomal subunit can affect mRNA-ribosomal interactions whereas binding to the large, 50S, subunit interferes with the peptidyl transferase activity [68]. In the absence of antibiotics it was expected that the quinary interactions of Trx would not vary over time, and because ribosome inhibitors can alter mRNA-ribosome interactions, Trx quinary interactions could be profoundly altered.

**Figure 7:**
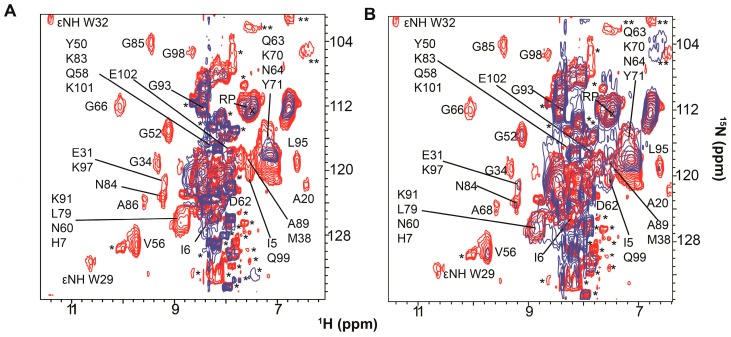

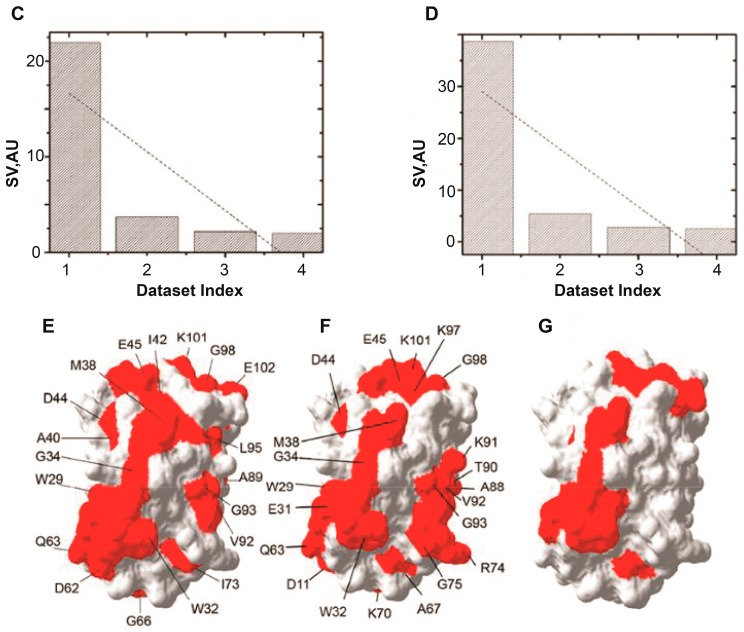
Binding of tetracycline and streptomycin to ribosomes changes the quinary structure of Trx in *E. coli*. (**A**) Overlay of the in-cell ^1^H-^15^N CRINEPT–HMQC–TROSY spectra of [*U*- ^15^N] Trx without (red) and with (blue) tetracycline. (**B**) Overlay of the in-cell ^1^H-^15^N CRINEPT–HMQC–TROSY spectra of [*U*- ^15^N] Trx without (red) and with (blue) streptomycin. Single and double asterisks indicate peaks from metabolites and unassigned side chain protons, respectively. The overlaid spectra are at the same contour levels. The reference peak used for peak intensity normalization is indicated by RP. (**C**) Distribution of singular values of each dataset index (binding mode) for Trx residues in the presence of tetracycline. (**D**) Distribution of singular values of each dataset index (binding mode) for Trx residues in the presence of streptomycin. (**E**) Residues involved in quinary interactions (red) due to the presence of tetracycline are mapped onto the molecular surface of Trx (Protein Data Bank entry 1X0B). (**F**) Residues involved in quinary interactions (red) due to the presence of streptomycin. (**G**) Quinary interaction surface (red) of Trx in the absence of antibiotics. Panels B–G are adapted from Breindel et al. (2017) [36].

Using a bioreactor that monitors real-time changes in in-cell NMR spectra, Breindel et al. [36] administered tetracycline and streptomycin, which bind to the 30S subunit, and chloramphenicol, which binds to the 50S ribosomal subunit, to *E. coli* containing overexpressed [*U*- ^15^N] Trx. The in-cell ^1^H-^15^N CRINEPT-HMQC-TROSY spectra were analyzed by using SVD to identify concentration-dependent changes as the concentration of antibiotic increased. The spectra were extensively broadened in the presence of tetracycline (Figure 7A) and streptomycin (Figure 7B). SVD analysis showed a sharp drop in the Scree plot of singular values with poor linear fits, r^2^ of 0.67 and 0.66 for tetracycline (Figure 7C) and streptomycin (Figure 7D) respectively, indicating specific changes in quinary interactions. Not unexpectedly, the addition of chloramphenicol, which does not disturb the binding of mRNA, resulted in a linear decrease in singular values, r^2^ = 0.94, suggesting that Trx quinary interactions were not perturbed.

Comparable changes in the Trx quinary interaction surface resulted from treating the cells with tetracycline and streptomycin respectively (Figure 7E,F). A large interaction surface containing negatively-charged and hydrophobic residues and a smaller patch containing positively-charged and hydrophobic residues are very similar to the Trx interaction surface in the absence of antibiotics (Figure 7G). A third adjoining surface, which does not participate in quinary interactions in the absence of antibiotics was differentially perturbed by tetracycline and streptomycin. In addition to surface residues, a number of buried residues underwent broadening in the presence of antibiotics, suggesting that some tertiary structural changes in Trx be occurring.

### 5.3. Dihydrofolate reductase and Thymidylate synthase

**Figure 8:**
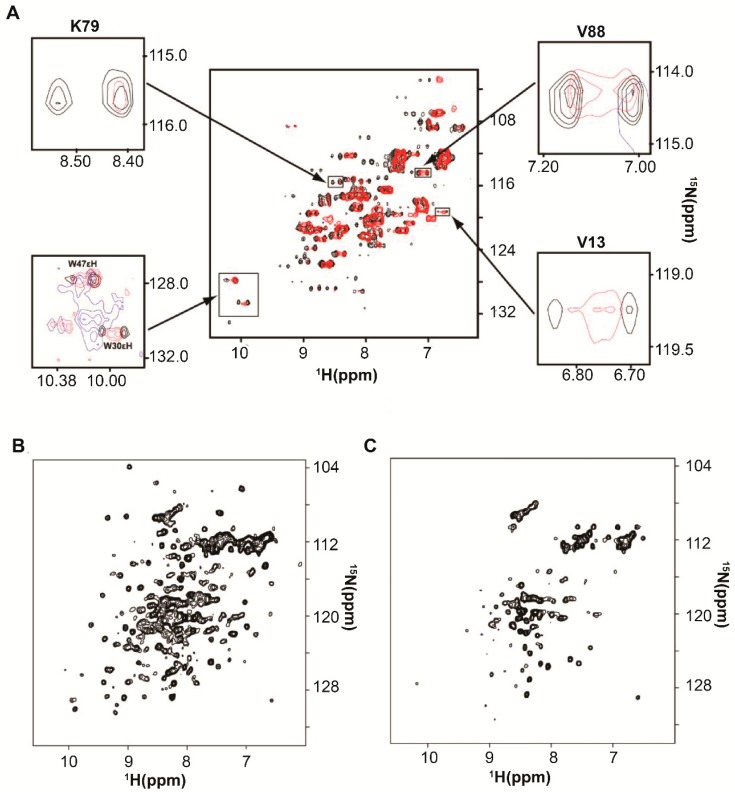
Dihydrofolate reductase, DHFR, and Thymidylate synthase, TS, engage in quinary interactions with RNA. (**A**) Overlay of *in vitro* ^1^H-^15^N CRINEPT–HMQC–TROSY spectra of 200 μM [*U*- ^2^H, ^15^N] DHFR with 0.5 mM folate (black) and with 0.5 mM folate and 2.5 μ M ribosome (red). Insets show individual residue overlays that include in-cell NMR peaks (blue). Folate was added to increase the solubility of DHFR. (**B**,**C**) ^1^H-^15^N HSQC spectra of 50 μΜ [*U-* ^15^N] TS with (**B**) 0 μg and (**C**) 135 μg of total *E. coli* RNA. The figure is adapted from DeMott et al. (2017) [26].

DeMott et al. 2017 [26] investigated two additional metabolic proteins: Dihydrofolate reductase [69,70], DHFR, and Thymidylate synthase [71], TS. The ^1^H-^15^N CRINEPT-HMQC-TROSY spectrum of DHFR was broadened in-cell and in vitro in the presence of ribosomes (Figure 8A). The ^1^H-^15^N HSQC in vitro NMR spectra of TS systematically broadened as the concentration of total *E. coli* RNA was increased (Figure 8B,C). Ribosomes were subsequently shown to affect the kinetic activity of TS. Thus both enzymes acquired quinary structure by interacting with ribosomes.

## 6. Ribosome-Mediated Regulation of Biological Activity

### 6.1. Adenylate Kinase

The studies delineated above showed that the quinary structures of ADK and Trx are mediated by protein-RNA interactions and that these structures can be affected directly and indirectly by perturbing the ribosome through the application of ribosomal-binding antibiotics. To investigate the possibility that the quinary state of the target protein may affect its biological activity, assays were performed in the presence of ribosome preparations.

**Figure 9:**
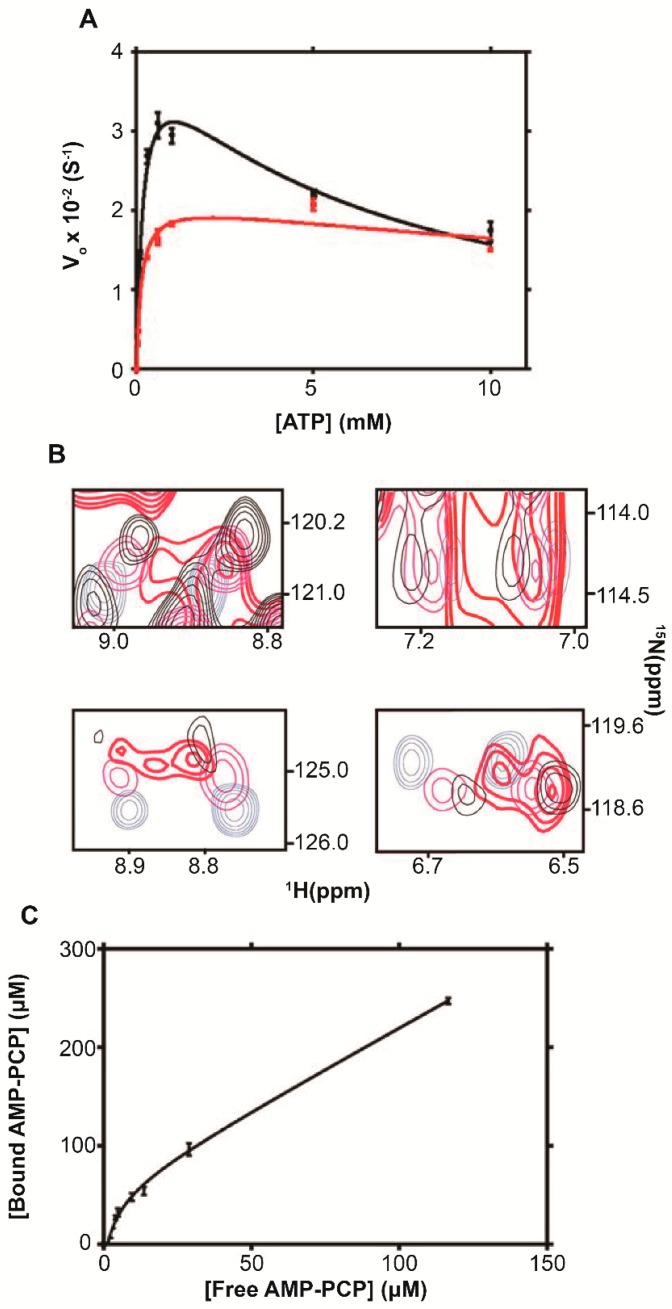
Ribosomes modulate ADK enzymatic activity. (**A**) Kinetic activity profile for ADK without (black) and with (red) 1 μM ribosome. (**B**) Overlays of in vitro ^1^H-^15^N CRINEPT–HMQC–TROSY spectra of 10 μM [*U*- ^2^H, ^15^N] ADK at 0 μM adenosine triphosphate, ATP, (blue), 20 μM ATP (magenta), 40 μM ATP (black), and 80 μM ATP plus 1 μM ribosome (red). (**C**) ATP analogue β,γ-methyleneadenosine 5′-triphosphate, AMP-PCP binding to ribosomes. The concentration of ribosomes was 2 μM. The figure is adapted from DeMott et al. (2017) [26].

DeMott et al., 2017 [26] found that in the absence of ribosomes the V_max_ for ADK reached a maximum at ∼1 mM and decreased at higher ATP concentrations, characteristic of noncompetitive substrate inhibition (Figure 9A). The kinetic profile suggests the presence of additional ATP binding sites [72,73]. Adding 1 μM ribosome decreased V_max_ by 50%, increased the substrate affinity by 30% and decreased the affinity of inhibitor binding, K_I_, 6-fold (Table 2). The interaction between ADK and the ribosome does not occlude the active sites (Figure 4) but may preclude occupancy of allosteric binding sites. This would be consistent with a reduction in binding affinity for a second ATP binding site exemplified by K_I_. Thus, the interaction between ADK and ribosomes establish a quinary activity state that reduces the V_max_ of ADK and mitigates substrate inhibition.

To assess the effect of ribosomes on the interaction between ATP and ADK, ^1^H-^15^N CRINEPT-HMQC-TROSY spectra of purified 10 μM [*U*- ^15^N] ADK were collected in the presence of increasing amounts of ATP. Systematic changes in the intensities and chemical shifts of interacting residues of ADK were observed as the concentration of ATP was increased from 0 to 40 µM. In the presence of 1 μM ribosome, the 80 μM ATP ^1^H-^15^N CRINEPT-HMQC-TROSY spectrum coincided more closely with the ADK spectrum acquired at 40 µ M ATP in the absence of ribosomes. The result suggests that the ribosome reduced the concentration of free ATP available for binding. This was consistent with the in-cell observation of an open conformation for ADK [14], which implied that only a small fraction of intracellular ATP binds to ADK, k_M_ = 51 µM [74], despite the fact that bacterial cells contain ~3 mM total ATP.

To investigate a possible mechanism for reducing the concentration of free ATP in *E. coli*, 2D ^1^H-^31^P-correlation NMR experiments were performed to quantify the binding of β,γ-methyleneadenosine 5′-triphosphate, AMP-PCP, a noncleavable ATP analogue, to ribosomes. The pH of the solution remained constant during the titration. Below 10 μM, the binding of AMP-PCP was fit to a single class of sites with an apparent K_d_ of 6 ± 2 μM; the inability to saturate the binding curve at higher concentrations prevented the estimation of an affinity constant for the weaker class of binding (Figure 9C). Thus it appears that ribosomes attenuate the in-cell activity of ADK by binding large amounts of ATP, thereby reducing the intracellular concentration of free ATP available to drive binding reactions, and suppress substrate inhibition through quinary interactions that reduce the affinity of regulatory sites.

### 6.2. Dihydrofolate Reductase and Thymidylate Synthase

**Figure 10:**
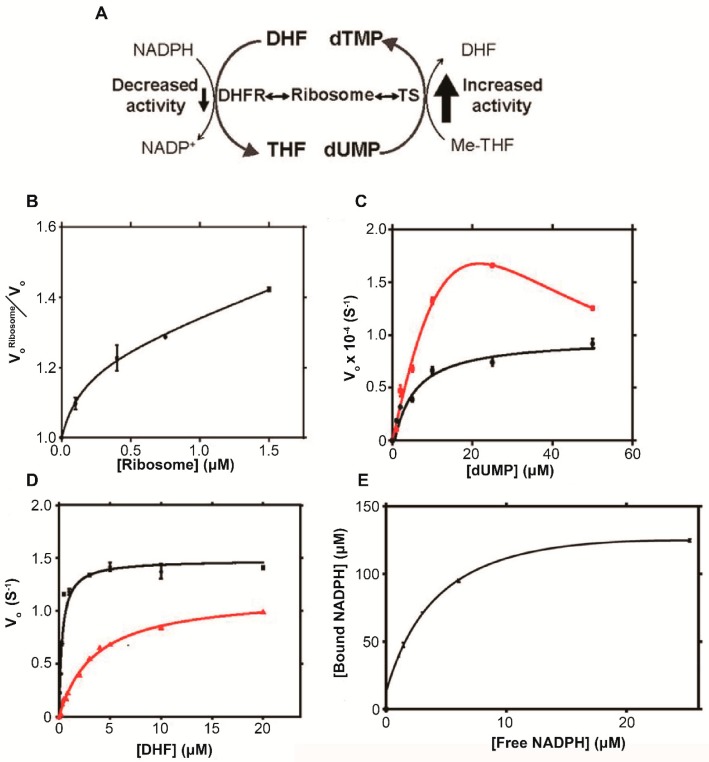
Ribosomes modulate TS and DHFR enzymatic activities. (**A**) Function linkage between TS and DHFR in the thymidylate synthetic pathway (**B**) Increase in TS activity with increasing ribosome concentration. (**C**) Kinetic activity profile for TS without (black) and with (red) 0.5 μM ribosome. (**D**) Kinetic activity profile for DHFR without (black) and with (red) 0.5 μM ribosome. (**E**) NADPH binding to ribosomes. The concentration of ribosomes was 1 μM. Figure is adapted from DeMott et al. (2017) [26].

TS and DHFR are functionally linked in the *de novo* thymidylate synthetic pathway (Figure 10A) [71,75]. TS catalyzes the conversion of dUMP to dTMP yielding dihydrofolate, DHF. DHFR uses the coenzyme NADPH to convert DHF, to tetrahydrofolate, THF, for the biosynthesis of purines, thymidylic acid and some amino acids. DeMott et al. 2017 [26] examined the effect of ribosomes on the activity of these enzymes.

The activity of TS increased with increasing ribosome concentration (Figure 10B). In the presence of 0.5 µM ribosomes V_max_ increased ~20-fold, substrate binding affinity decreased ~20-fold and a K_I_ of 3.9 ± 0.5 µM was resolved (Table 2). The kinetic profile was characteristic of uncompetitive substrate inhibition in which TS-ribosome quinary interactions increased the catalytic rate and promoted substrate inhibition (Figure 10C). In the presence of 0.5 μM ribosomes, DHFR displayed a ∼20% decrease in V_max_ and a 10-fold decrease in substrate binding affinity and a kinetic profile consistent with the ribosome acting as a competitive inhibitor (Table 2; Figure 10D). The reduced activity may be due to the DHFR-ribosome interface blocking or altering DHF and/or NADPH binding sites, and/or NADPH binding to ribosomes lowering the concentration of free NADPH available for DHFR catalysis. Indeed, NADPH was shown to bind specifically to ribosomes with a dissociation constant of 4.5 ± 1.5 µM (Figure 10E).

The results suggest a possible mechanism through which ribosome-mediated quinary structural interactions act to reduce cellular levels of dUMP (Figure 10A). Ribosome suppression of DHFR activity lowers the intracellular concentration of THF, which is converted into Me-THF. The decrease in Me-THF concentration reduces the ability of TS to utilize the substrate resulting in a buildup of dUMP (Figure 10A). However, ribosomal-dependent enhancement of TS activity (Figure 10B) increases the catalytic rate allowing the mutagenic substrate to be metabolized. This shows the potential for ribosomes to regulate cellular processes through compensatory adaptations of functional linkages.

## 7. Discussion

The cytosol of an *E. coli* cell is highly congested containing about 300 mg/mL of macromolecules [76]. Such a high concentration creates an enormous excluded volume through macromolecule crowding, which in turn reduces the concentration of bulk water while simultaneously increasing the concentration of macromolecular and ionic species. The reduced water activity affects equilibria governing hydrophobic and hydrophilic interactions and the solvent shells on protein surfaces. The increase in soluble species, in combination with intermolecular distances less than the typical Debye radius for ion charges [77], i.e., ~0.7 nm, reduce the effects of electrostatic screening, promote electrostatic interactions and inevitably increase the propensity for transient low-affinity interactions.

In this review, we summarized work that identified transient low-affinity protein–RNA interactions, historically called quinary [18] by using in-cell ^1^H-^15^N CRINEPT-HMQC-TROSY NMR spectroscopy to overcome the effects of extreme broadening of spectral crosspeaks. Quinary structures are large transient complexes that affect protein stability [78,79] and can modulate ligand binding and protein function. Similar interactions have been detected in highly concentrated cell lysates [16,80,81]. The effects of RNA on peak broadening were reconstituted in vitro using preparations of total RNA from both prokaryotic and eukaryotic cells, and purified ribosomes thus confirming the specificity of the interactions [14,26].

The initial observation of extreme crosspeak broadening in in-cell NMR spectra aspired in vitro studies to attribute the phenomenon to the effects of excluded volume, macromolecular crowding and increased intracellular viscosity [15,16,17,82,83,84]. These studies provided useful insight into physical mechanisms for limited spectral broadening but none were able to fully reproduce the effects seen in-cell. The binding of a labeled target protein to a large cellular component is the driving force behind spectral broadening due to the reduction in tumbling rate that accompanies the massive increase in the apparent molecular size of the target, which in turn, affects the magnitude of the NMR signal. These interactions underlie the quinary protein structure and can have a profound influence on the activity of the target and its regulation.

Most cytosolic proteins exhibit activity in the absence of other macromolecular species, i.e., in vitro, requiring only substrates and co-factors. Indeed, observations made under these conditions have provided the basis for understanding and modeling much cellular physiology and metabolism. In-cell the activity resulting from metabolic enzymes and other cytosolic species engaging in quinary interactions originates from a population of free and bound species and their derivative functional linkages. In addition, the increase in ribosome concentration with cell growth [85,86] further modulates the distribution between free and bound protein. Thus, the effect of the ribosome on the net activity is to fine-tune and regulate the metabolism of the cell both directly, as is the case for ADK-ribosome interactions, or indirectly, as shown for Trx-mRNA-ribosome interactions in growing cells.

The micromolar concentration of ribosomes in prokaryotes and eukaryotes [24] virtually assures that the ribosomal-binding interactions described, all of which exhibit micromolar dissociation constants, occur inside live cells. Accordingly, we propose that the ribosome plays a role in organizing metabolism [87] by serving as a hub for concentrating enzymes and metabolites. In actively growing *E. coli*, the fractional volume occupied by fully processed 70S ribosomes is ~0.16 [24] and may increase up to four times, ∼0.64, outside the space occupied by the nucleoid [88,89,90]. When compared to the fraction of space occupied by closely packed hard spheres, 0.74, [91] this implies that *E. coli* ribosomes are tightly packed in the cytosol with the volume available for biological reactions restricted to the “free” spaces delimited by ribosome surfaces [92]. In this manner, the surfaces of the ribosome become the operational milieu for much biological activity. Going forward, further in-cell NMR spectroscopy and models of cellular metabolism that depend on activity gleaned in vitro must consider the inescapable effects of ribosomes on these processes.

## Figures and Tables

**Table 1:** Summary of protein quinary interactions.

Protein	Binds	Effect of RNA-Binding
Ubiquitin (Ubq)	Total RNA [14,35] mRNA [28]	Blocks polyubiquitination sites, increases apparent MW [14]
Thioredoxin (Trx)	Total RNA [14], mRNA [28]	Increases apparent MW [14] Antibiotic binding to ribosome alters quinary structure [36]
Adenylate kinase (ADK)	Total RNA [14] mRNA [28] Ribosome [26,29]	Increases apparent MW [14] Noncompetitive kinetic inhibitor [26]
Dihydrofolate reductase (DHFR)	mRNA [37,38] Ribosome [26]	Competitive kinetic inhibitor [26]
Thymidylate synthase (TS)	Total RNA [26] mRNA [28,39] Ribosome [26,29]	Uncompetitive kinetic activator [26]

**Table 2:** Kinetic Parameters Resolved for ADK, DHFR and TS in the Absence and Presence of Ribosomes.

Enzyme	[ribosome] (μM)	V_max_ (S^−1^)	V_max_^Ribosome^/V^o^_max_^a^	K_M_ (μM) ^b^	K_I_ (mM) ^b^	R^2^
ADK	0	(4.2 ± 0.2) × 10^−2^	∼0.5	180 ± 20	6.1 ± 0.8	0.98
	1	(2.1 ± 0.1) × 10^−2^		130 ± 30	35 ± 2	0.96
DHFR	0	(1.48 ± 0.04) × 10^−4^	∼0.8	0.32 ± 0.04		0.95
	0.5	(1.16 ± 0.02) × 10^−4^		3.5 ± 0.1		0.99
TS	0	(9.7 ± 0.4) × 10^−5^	∼20	5.4 ± 0.7		0.97
	0.5	(2.0 ± 0.2) × 10^−3^		120 ± 20	(3.9 ± 0.5) × 10^−3^	0.99

^a^ V_max_^Ribosome^ and V_max_ are the maximum initial velocities with and without the ribosome. ^b^ Enzymatic parameters in the absence of ribosomes are consistent with those found at http://www.brenda-enzymes.org. Table adapted from DeMott et al. (2017) [26].
